# Design and Experimental Validation of a Synchronized Multi-Rate Wireless Sensor Network for Multimodal Physiological and Biomechanical Data Acquisition

**DOI:** 10.3390/s26216822

**Published:** 2026-10-28

**Authors:** Ali Pourhossein, Charmayne M. L. Hughes

**Affiliations:** Age-Appropriate Human-Machine Systems, Institute of Psychology and Ergonomics, Technische Universität Berlin, 10623 Berlin, Germany; pourhossein@tu-berlin.de

**Keywords:** multimodal data acquisition, multi-rate sensing, wireless sensor network, clock synchronization, data integrity, ESP-NOW

## Abstract

Reliable multimodal measurement requires coordinated acquisition of physiological and biomechanical signals operating at different sampling rates. A six-node wireless architecture was developed to support seven data streams: three surface electromyography (sEMG) signals (2 kHz each), photoplethysmography (PPG, 400 Hz configured), inertial measurements (416 Hz configured), and independent pinch- and grip-force streams (nominal 80-SPS HX711 mode; measured at 85.1 and 85.9 samples/s, respectively). An ESP32-S3 gateway uses a single ESP-NOW network for sensor-data delivery and continuous per-node clock compensation while preserving raw measurements, sample-level timing metadata, and end-to-end integrity diagnostics. Across three independent 30-min full-system recordings, pooled packet delivery and sample continuity were 99.987% and 99.988%, respectively. External common-input validation between two independent sEMG nodes yielded a drift-compensated timing mean absolute error of 0.0754 ms across 897 accepted events. A separate six-node GPIO reference-pulse test yielded a node-clock alignment mean absolute error of 0.3123 ms across 5319 matched events with 99.49% of events below 1 ms. Network-load and indoor distance tests further characterized communication robustness. These results demonstrate the feasibility and reliability of a unified wireless architecture that integrates heterogeneous multi-rate sensing, continuous clock-drift compensation, preservation of original sensor measurements, and comprehensive data-integrity monitoring for synchronized physiological and biomechanical data acquisition.

## 1. Introduction

Wearable and distributed sensing systems increasingly combine multiple physiological and biomechanical modalities to provide a more complete representation of human function than can be obtained from a single modality in isolation. This is particularly important because functional behavior emerges from interactions between neural, muscular, cardiovascular, and mechanical processes rather than any one system alone. Signals including surface electromyography (sEMG), photoplethysmography (PPG), inertial measurement units (IMUs), and interaction forces provide complementary information relating to muscular activation, cardiovascular dynamics, movement, and physical performance. Multimodal wearable sensing has therefore become an important direction in health monitoring, biomechanics, rehabilitation, ergonomics, and human–machine interaction [1,2,3,4].

A central difficulty in distributed wearable sensing is that different modalities impose substantially different acquisition requirements. sEMG generally requires substantially higher sampling rates than slowly varying force signals, while optical and inertial sensors may operate at intermediate rates. Imposing a single unified sampling rate across all the sensing elements would either oversample lower-bandwidth modalities (e.g., force and inertial sensors), increasing data volume and wireless traffic, or undersample higher-bandwidth signals (such as sEMG) and compromise temporal fidelity. These heterogeneous sampling requirements are further complicated when the sensing modalities are distributed across physically independent wireless nodes. Such nodes possess independent oscillators whose relative clock offsets and frequency errors accumulate over time. Consequently, timestamps based only on local node clocks or host-side packet-arrival times are insufficient when the intended analysis requires temporal relationships between signals acquired at different body locations.

Several wireless sensing platforms have addressed distributed physiological and biomechanical measurement [5,6,7,8,9], although individual approaches typically emphasize specific aspects of wireless acquisition, synchronization, or sensing integration. Biagetti et al. developed a Bluetooth Low Energy (BLE) device that is capable of acquiring multiple bioelectrical channels and inertial signals [5]. Serrani and Aliverti characterized wireless communication performance in a wearable monitoring platform by evaluating transmission behavior under different communication conditions and distances [6]. Sondej and Bednarczyk proposed a high-accuracy synchronous wireless sensor-network architecture based on a proprietary TDMA protocol, with an emphasis on sampling synchronization and ultra-low-power operation [7]. Biagetti et al. investigated high-accuracy clock synchronization in wireless sEMG sensors and demonstrated the importance of compensating timing errors produced by independent clock domains and wireless communication [8]. Li et al. developed an application-layer synchronization and data-alignment method for distributed BLE biosignal sensors and validated inter-node timing using common-input signals [9].

Beyond synchronization-focused and modality-specific wireless systems, other platforms have addressed the integration of heterogeneous sensing modalities within a common acquisition architecture. PhysioEdge combines multiple biomedical sensing modalities with multi-node synchronization and centralized wireless aggregation [10]. In parallel, because high-rate biosignal streams place substantial demands on wireless throughput, communication stability, and latency, work on the wireless communication layer remains directly relevant to multimodal wearable acquisition. Inafuco et al. [11] presented the MOT system, which used ESP-NOW for six-channel wireless sEMG acquisition and characterized analog performance, communication distance, packet loss, latency, and power consumption. Although MOT was developed for a single high-rate biosignal modality, it highlights the importance of communication-layer characterization for wireless acquisition systems that must preserve signal continuity under bandwidth and latency constraints.

Related ESP-NOW-based work has also examined multi-node timing and telemetry performance in lower-rate sensing contexts. Smelcerz [12] investigated an ESP32/MicroPython edge–cloud sensor network using ESP-NOW communication, gateway-referenced synchronization, and up to five environmental sensing nodes. This provides a useful comparison point for ESP-NOW-based synchronization, but the sensing and acquisition demands differ from those of multimodal physiological systems. That work focused primarily on packet-level latency decomposition in low-rate edge–cloud telemetry, whereas the present platform targets continuous heterogeneous physiological and biomechanical acquisition with modality-specific rates up to 2 kHz and continuous per-node clock-rate compensation. Taken together, these studies demonstrate the feasibility of distributed wireless sensing and multi-node timing coordination while differing substantially in sensing scope, radio architecture, synchronization strategy, acquisition rate, and validation methodology. Table 1 provides a quantitative and architectural comparison with representative published wireless sensing systems, including physical node count, sensing modalities, sampling rates, communication protocols, synchronization methods, experimentally reported timing performance, data transmission, power or operating autonomy, and evaluated communication range.

As shown in Table 1, the proposed platform is distinguished by the coordinated integration of heterogeneous physiological and biomechanical sensing, continuous clock-rate compensation, and multi-layer data-integrity monitoring within a single ESP-NOW network. While MOT [11] demonstrates six-node wireless sEMG acquisition, the present architecture additionally integrates PPG, IMU, and independent pinch- and grip-force measurements operating at different sampling rates. Compared with PhysioEdge [10], which employs a dedicated synchronization radio, the proposed system supports both sensor-data transmission and clock-synchronization exchanges through the same wireless communication layer. Another distinguishing capability is the preservation of original sensor measurements and sample-level timing metadata alongside packet-, sample-, synchronization-, and host-transport diagnostics. These capabilities were evaluated together through three independent 30-min full-system recordings and complementary external clock-alignment experiments. The principal contribution therefore lies in the experimentally validated combination of these functionalities rather than a new fundamental synchronization algorithm or superiority in any individual performance metric.

The complete system comprises six independently powered wireless physical nodes producing seven data streams: three independent sEMG streams, red and infrared PPG, a six-axis IMU, pinch force, and grip force. Rather than forcing all the sensors into a common sampling frequency, each subsystem retains its native or application-specific rate. Sensor measurements are associated with node-local timing information, and a central gateway maintains a continuously updated mapping between each node clock and a common master time base.

The principal contributions of this work are: (1) a heterogeneous six-node wireless architecture supporting seven physiological and biomechanical streams with modality-specific acquisition rates; (2) a two-way per-node synchronization method with continuous clock-rate compensation and mapping to a common master time base; (3) end-to-end packet-, sample-, synchronization-, and transport-level integrity monitoring from sensing nodes to host software; and (4) experimental system-level validation covering sustained full-load operation, network loading, communication distance and obstruction, and external inter-node timing accuracy.

This study focuses on system-level acquisition validation rather than clinical validation of each sensing modality. Accordingly, the commercially available PPG and inertial sensing elements are evaluated primarily as components of the synchronized acquisition architecture. Likewise, the force devices are incorporated as wireless biomechanical nodes, while transducer-specific metrological validation is outside the scope of the present work. The evaluation therefore emphasizes timing, continuity, communication performance, and sustained heterogeneous operation of the complete acquisition system.

## 2. Materials and Methods

### 2.1. Overall System Architecture

The system follows a star topology consisting of six wireless sensor nodes, one ESP32-S3 (Espressif Systems, Shanghai, China) central gateway, and a host computer running a custom graphical application. All the sensor nodes are independently battery-powered and transmit measurements directly to the central gateway using ESP-NOW on a common 2.4 GHz wireless channel. The gateway aggregates incoming streams, estimates the temporal relationship between each physical node clock and the gateway clock, converts node-local timestamps into a common master time base, and transfers binary frames to the host computer through native USB. The host application provides real-time visualization, synchronized data recording, synchronization and communication diagnostics, and session-level acquisition control.

Figure 1 summarizes the star topology and relationship between the sensor nodes, central gateway, and host application. The topology comprises six physical wireless nodes: Node 1 for EMG1, Node 2 for PPG and IMU, Node 3 for pinch force, Node 4 for grip force, Node 5 for EMG2, and Node 6 for EMG3. The node numbering follows the implemented firmware assignments and is retained consistently throughout the recorded datasets and system diagnostics. Among these nodes, Node 2 is the only unit that contains two sensing modalities. The PPG and IMU streams therefore share the same ESP32-C3 node-local timestamp domain and node-to-master synchronization model, although the MAX30102 and LSM6DS3 retain independent sensor-side acquisition engines and sampling schedules. Their measurements are acquired and packetized independently before transmission from the shared wireless node. The remaining sensing modalities are implemented as physically independent wireless units. During operation, the gateway identifies each sensor node from its first valid data packet, records its MAC address, adds the device as an ESP-NOW peer, and subsequently includes it in the synchronization process.

Wireless communication is implemented using ESP-NOW on 2.4 GHz Wi-Fi channel 1. This connectionless protocol enables direct sensor-to-gateway communication without a conventional IP connection [13]. Because ESP-NOW v1 limits the application payload to 250 bytes, modality-specific packets were constrained to this size. Peer encryption was disabled, and the application did not explicitly override the radio transmission power or ESP-NOW physical-layer data rate. The three EMG nodes used the same acquisition and transmission architecture with distinct node identifiers. Source sequence numbers and queue-drop counters permitted separate stream-level communication diagnostics.

Despite these modality-specific packet formats, all the source packets share a common header to support stream identification, temporal reconstruction, and integrity monitoring at the gateway. This header includes the protocol magic value and version, physical node ID, sensor type, flags, sample count, packet sequence number, first sample index, local timestamp of the first sample, and nominal sampling period. Sensor-specific measurements and diagnostic fields are appended after the common header. The relationship between the packet-level first-sample timestamp and the underlying sensor acquisition timing is modality-dependent and described in Table 2. Sequence counters allow the gateway to identify missing, duplicated, and out-of-order packets; duplicated or late packets are recorded diagnostically and are not forwarded as new sensor data.

Based on the packet rates summarized in Table 2, the three EMG streams generate a combined 300 source packets/s under nominal operating conditions, while the PPG and IMU streams generate 25 and approximately 29.7 packets/s, respectively. The two force nodes of the MeRKeL wireless pinch-and-grip dynamometer [14] together contribute approximately 32 packets/s. The resulting aggregate nominal source traffic is therefore approximately 386.7 packets/s excluding synchronization exchanges.

#### Embedded Firmware Configuration and Task Scheduling

The embedded implementation uses the Arduino framework for ESP32 together with FreeRTOS tasks and queues. The ESP32-S3 gateway receives ESP-NOW frames through a Wi-Fi receive callback, timestamps them using the ESP microsecond timer, and places them into a 128-frame receive queue. Packet processing, node discovery, and synchronization scheduling are performed in the Arduino main loop. A separate USB transmission task with FreeRTOS priority 4 transfers binary frames to the host through a 48-frame queue. The sensing nodes separate acquisition, synchronization-response processing, and wireless transmission into dedicated tasks. Table 3 summarizes the task priorities and queue capacities defined in the available firmware revisions.

All the embedded firmware was developed using Arduino IDE version 2.3.3 (Arduino S.r.l., Monza, Italy) and the Espressif Systems ESP32 board package version 3.3.12. The implemented firmware revisions were gateway v3.2 (CRC Fix), EMG v4 for all three EMG nodes, PPG/IMU v6, and force-node v2 for both pinch and grip devices. The PPG/IMU implementation uses the SparkFun MAX3010x library (SparkFun Electronics, Niwot, CO, USA) for optical sensing and SparkFun LSM6DS3 library version 1.0.3 for inertial measurements. The wireless experiments were conducted with all the devices operating on Wi-Fi channel 1. These software configurations correspond to the implementation evaluated in this study.

### 2.2. Surface Electromyography Nodes

Three identical battery-powered sEMG units were developed as independent wireless nodes. The units are designated EMG1, EMG2, and EMG3, corresponding to Node IDs 1, 5, and 6, respectively, and use identical hardware and acquisition firmware. Each unit comprises a custom analog front-end, an 18-bit ADS8691 successive-approximation analog-to-digital converter (ADC; Texas Instruments, Dallas, TX, USA), and an ESP32-C3 microcontroller. Surface signals are acquired using a three-electrode arrangement comprising two differential measurement electrodes and one reference electrode.

The overall signal-conditioning architecture follows established sEMG acquisition practice in which differential amplification, common-mode rejection, controlled bandwidth, low noise, and sufficient sampling resolution are important [15]. The analog front-end is based on an INA128 instrumentation amplifier followed by active conditioning stages implemented with OPA2134 operational amplifiers (both from Texas Instruments, Dallas, TX, USA). The custom circuit incorporates separate analog and digital power domains and provides bipolar analog supply rails required by the front-end. The ADS8691 provides 18-bit SAR conversion with programmable bipolar input ranges and an SPI-compatible digital interface [16,17,18]. The implemented custom sEMG hardware is shown in Figure 2. The analog conditioning stages were designed to provide a nominal mid-band voltage gain of approximately $2.9\times 10^{3}$ (69.3 dB) with an estimated −3 dB frequency range of approximately 22–473 Hz.

Each sEMG device is powered by two 3.7 V, 750 mAh lithium-polymer cells connected in series, providing a nominal pack voltage of 7.4 V. The ADC is read using SPI at 1 MHz. Sampling is scheduled at 2000 Hz, corresponding to a nominal inter-sample interval of 500 μs. Twenty samples are accumulated in each wireless packet, resulting in a nominal packet-generation interval of 10 ms. To preserve the actual local acquisition timing, the timestamp assigned to each sEMG sample corresponds to the recorded local start time of the ADS8691 conversion that produced that sample. The conversion-start time is captured from the ESP32-C3 microsecond timer at the chip-select rising edge, while the 2 kHz hardware timer schedules successive ADC reads at nominal 500 μs intervals. Each packet stores the local timestamp of its first sample together with relative microsecond timestamps for the subsequent samples so that sample times are not reconstructed solely from the nominal 2 kHz rate. The 18-bit ADC measurements are centered around zero prior to transmission but are otherwise preserved without signal-domain filtering at the node.

To support continuous high-rate acquisition during temporary wireless communication failures, the EMG firmware implements a dedicated transmission queue with a capacity of 12 packets and a link-recovery mechanism. Following five consecutive transmission failures, the node enters a link-down state while maintaining its 2 kHz acquisition schedule. During this state, outdated packets are discarded and the most recent completed packet is periodically used to probe communication recovery at 500 ms intervals. Once transmission succeeds, normal packet delivery resumes. This approach prioritizes the availability of recent measurements and prevents stale data from accumulating during communication interruptions.

### 2.3. PPG and IMU Node

Node 2 integrates both PPG and inertial sensing within a single wireless unit. Optical measurements are acquired using a MAXREFDES117# reference module based on the MAX30102 optical sensor (Analog Devices, Wilmington, MA, USA). MAXREFDES117 integrates red and infrared optical channels for heart-rate and pulse-oximetry development [19]. The PPG module is connected to the ESP32-C3 of Node 2 through I^2^C.

PPG acquisition is configured at 400 samples/s. The sensor operates with red and infrared channels, no sample averaging, a pulse width of 411 μs, and an ADC range setting of 16,384. Sixteen paired red/infrared measurements are grouped into each PPG packet. Unlike the sEMG nodes, PPG sampling is generated by the MAX30102 internal acquisition clock rather than an ESP32-C3 software or hardware timer. The firmware polls the hardware FIFO at approximately 1 ms intervals. When unread samples are detected, the node-local ESP32-C3 microsecond timer is recorded when the FIFO state is observed and used as the timing anchor for the newest available sample. If multiple samples are retrieved in one FIFO service operation, earlier samples in that batch are timestamped by stepping backward from this anchor using the configured 2.5 ms sampling interval. The resulting sample times are stored as relative microsecond offsets within the wireless packet. To preserve acquisition-state information, the firmware accesses the MAX30102 hardware FIFO directly; FIFO rollover is disabled so that overflow conditions remain detectable rather than silently overwriting unread samples. Raw optical measurements are transmitted without median filtering, signal inversion, thresholding, or floating-point conversion. The PPG data are therefore treated as raw optical acquisition data rather than pre-computed heart-rate or oxygen-saturation estimates.

IMU data are acquired using a DollaTek breakout module containing an LSM6DS3 (STMicroelectronics, Geneva, Switzerland) six-degree-of-freedom (DoF) sensor, which integrates a three-axis accelerometer and three-axis gyroscope with programmable output data rates and measurement ranges [20]. The IMU shares the ESP32-C3 and I^2^C bus of Node 2 with the PPG module but is sampled and packetized independently. The accelerometer and gyroscope are configured at 416 Hz, the closest native output data rate to the 400 Hz PPG acquisition rate. The accelerometer range is ±16 g and the gyroscope range is ±2000 deg/s. As with the PPG, IMU sampling is generated by the LSM6DS3 internal output-data-rate clock. The node polls the sensor status register at approximately 1 ms intervals. When new accelerometer and gyroscope data are both available, a node-local microsecond timestamp is captured immediately before the six-axis register burst is read. Each accepted measurement retains the local time at which the availability of the new sensor sample was detected. Fourteen six-axis measurements are grouped into each IMU packet while retaining the sensor-native 416 Hz acquisition rate without resampling on the embedded node. The first local timestamp and the relative microsecond offsets of the individual measurements are transmitted with the packet.

### 2.4. MeRKeL Pinch- and Grip-Force Nodes

The pinch- and grip-force streams are provided by the previously developed MeRKeL wireless dynamometer system, whose mechanical design, force-sensing configuration, calibration procedure, and transducer-specific laboratory and human feasibility evaluation have been reported previously [14]. In the present platform, the pinch and grip devices operate as two independent battery-powered wireless force nodes and are assigned Node IDs 3 and 4, respectively. Both units employ custom electronics based on an ESP32-C3 and an HX711 (Avia Semiconductor, Xiamen, China) 24-bit bridge ADC. The HX711 is operated on Channel A at a gain of 128 and with the RATE input configured for nominal 80 samples/s operation [21].

The force-node firmware follows the HX711 data-ready signal rather than imposing a software sampling timer. For each conversion, the ESP32-C3 local microsecond timer is sampled when the HX711 DATA-READY state is detected before the 24-bit conversion result is shifted from the device. The DATA-READY state is polled at approximately 1 ms intervals, and the resulting local timestamp is retained for each force sample. Five signed 24-bit raw ADC measurements are accumulated in each wireless packet. The packet stores the timestamp of the first force sample together with the measured relative microsecond offset of each subsequent sample; consequently, the recorded timing follows the realized HX711 conversion pacing rather than assuming a fixed 12.5 ms interval.

No tare, calibration scaling, sign inversion, or signal thresholding is applied on the embedded node. Instead, raw counts are transmitted to the PC together with timing information, source-error diagnostics, transmission-queue drops, and battery voltage. A one-second startup period is discarded to allow the ADC and bridge signal to stabilize. Force calibration is applied at the desktop level using device-specific calibration coefficients and a session-specific unloaded zero value. Raw HX711 measurements are retained regardless of the calibrated force output, allowing calibration models to be revised without loss of the original signal.

### 2.5. Central Gateway and USB Data Transport

The central gateway is based on an ESP32-S3-DevKitC development board and communicates with the host through the ESP32-S3 native USB CDC serial interface; no external UART or USB-to-UART bridge is used [22]. The gateway receives wireless packets, verifies packet type and sequence continuity, updates per-stream communication statistics, maps node-local timestamps into the common master time domain, and emits compact binary frames to the PC through native USB. The host serial API is configured with a nominal 921,600-baud parameter for interface compatibility; because data are transferred through native USB CDC, this value does not represent a physical UART line rate. Three frame classes are used for gateway-to-host transfer: sensor data, synchronization status, and master status. For sensor data frames, the gateway transfers the original source timing information together with the synchronized master time representation. A USB-level sequence number enables detection of missing host-transfer frames, and a 32-bit cyclic redundancy check (CRC-32) provides an independent end-to-end integrity check between the gateway and PC.

### 2.6. Wireless Clock Synchronization

Because each wireless node operates from an independent oscillator, local timestamps cannot be directly compared across devices. The central gateway therefore maintains an independent clock model for every wireless node. Synchronization uses a two-way four-timestamp exchange implemented at the application layer via the microsecond-resolution ESP timer on the gateway and sensor nodes. The gateway records $t_{1}$ immediately before passing the synchronization request to esp_now_send(). On the sensor node, $t_{2}$ is captured in the ESP-NOW receive callback before the request is queued for synchronization processing. After the node obtains exclusive access to the ESP-NOW transmitter, $t_{3}$ is captured immediately before the synchronization response is passed to esp_now_send(). Finally, the gateway records $t_{4}$ in its ESP-NOW receive callback before the returned frame is queued for subsequent processing. The exchange is illustrated in Figure 3.

These timestamps correspond to software-level ESP-NOW send and receive points rather than hardware MAC-layer transmission and reception instants. Accordingly, the synchronization model assumes that the residual forward and reverse communication delays are approximately symmetric after excluding exchanges with elevated round-trip time (RTT).

Node-side processing time is removed from the measured round-trip interval according to

(1) $$RTT=\left(t_{4}-t_{1}\right)-\left(t_{3}-t_{2}\right).$$

Synchronization responses with an RTT greater than 10 ms are excluded from the clock model. The gateway maintains cumulative per-node synchronization diagnostics including requests sent, responses received, rejected responses, and request-send errors, which are transferred to the host application. The corresponding midpoint times are calculated as

(2) $$t_{M}=\frac{t_{1}+t_{4}}{2},$$

and

(3) $$t_{N}=\frac{t_{2}+t_{3}}{2},$$

where $t_{M}$ represents the corresponding master-clock time and $t_{N}$ represents node-local time. The estimated instantaneous node-to-master offset is

(4) $$\hat{∆t}=t_{N}-t_{M}.$$

Let $∆t_{\mathrm{true}}$ denote the true node-to-master clock offset at the exchange midpoint, and let $d_{f}$ and $d_{r}$ denote the forward and reverse communication delays between the software timestamping points excluding the measured node-side processing interval. Assuming an approximately constant relative clock offset during an individual exchange, the estimated offset can be expressed as

(5) $$\hat{∆t}=∆t_{\mathrm{true}}+\frac{d_{f}-d_{r}}{2}.$$

Accordingly, delay asymmetry introduces an offset bias equal to half the difference between the forward and reverse delays. The synchronization timestamps are obtained using microsecond-resolution ESP timers; under an ideal timestamp-quantization model, the corresponding midpoint-offset uncertainty is bounded by approximately 1μs. However, software scheduling, transmission buffering, and wireless channel access may introduce larger timing variations. The RTT filter reduces the influence of exchanges with elevated communication delays but cannot independently identify or completely eliminate forward/reverse delay asymmetry.

To account for relative clock drift and its accumulated effect over time, the gateway uses a continuously updated linear clock model rather than relying on a single constant-offset estimate. For each wireless node, the gateway retains up to 32 recent synchronization observations. Within this rolling window, the observation with the lowest RTT serves as an adaptive reference for filtering. Observations are retained if their RTT is no more than 3.0 ms above this reference value subject to an absolute acceptance ceiling of 10 ms. At least four valid observations are required before the clock model is considered valid.

The 32-observation window was selected to balance the stability of clock-rate estimation with responsiveness to changes in oscillator drift. A shorter window provides a more recent estimation history but is more sensitive to individual synchronization observations, whereas a longer window provides greater smoothing but may reduce responsiveness. The 3.0 ms RTT margin was selected to limit the influence of delayed synchronization exchanges while retaining sufficient observations for reliable linear regression. The suitability of these parameters was subsequently examined through a dedicated sensitivity analysis.

A least-squares linear mapping is then estimated as

(6) $$t_{master}=t_{master,ref}+a\left(t_{node}-t_{node,ref}\right),$$

where $t_{\mathrm{node},\mathrm{ref}}$ and $t_{\mathrm{master},\mathrm{ref}}$ are the mean node- and master-domain midpoint times of the synchronization observations retained in the current regression window, respectively, and *a* is the least-squares slope mapping node-local time to the master time domain. The slope is computed from the centered synchronization observations as

(7) $$a=\frac{\sum_{k=1}^{n}\left(x_{k}-\bar{x}\right)\left(y_{k}-\bar{y}\right)}{\sum_{k=1}^{n}{\left(x_{k}-\bar{x}\right)}^{2}},$$

where $x_{k}$ and $y_{k}$ denote the node- and master-domain midpoints of the *k*-th retained observation, respectively, and *n* is the number of observations included in the regression. A model is rejected when fewer than four observations remain, the centered node-time variance sum is below $1\;\mu s^{2}$, or the slope is outside 0.9995–1.0005 (a ±500 ppm plausibility interval). The fit RMS represents the residual RMS of the retained observations rather than an independent measure of synchronization accuracy.

The fitted slope is used to express clock drift as

(8) $$D_{\mathrm{ppm}}=\left(\frac{1}{a}-1\right)10^{6}.$$

A positive value indicates a node clock running faster than the master. Local timestamps are subsequently mapped to the master clock using Equation (6).

The synchronization scheduler uses a 120 ms global pacing slot and a minimum per-node synchronization eligibility interval of 850 ms. Active nodes are considered in round-robin order, with at most one synchronization request transmitted during each scheduler slot. Because request timing is quantized by the 120 ms scheduler period, operation with all six active nodes results in approximately one synchronization request per node per second. Nodes that do not transmit valid data for 3 s are marked inactive. Large clock discontinuities and backward local-clock steps are treated as a new clock epoch and cause the synchronization model to be rebuilt. Under this scheduling scheme, synchronization exchanges are interleaved with normal sensor-data traffic, and sensor acquisition is not intentionally suspended while synchronization requests and responses are processed.

The gateway sets the packet-level synchronization-valid flag when the corresponding node is active, the current regression fit is valid, and the packet-reference time maps to a nonnegative master time. This flag does not impose a numerical threshold on fit RMS, model age, or independent clock-alignment error.

If an individual synchronization response is delayed beyond the RTT acceptance limit, rejected, or temporarily unavailable, the gateway continues to use the previously accepted clock model provided that the node remains active and the model remains valid. The age of the most recent accepted synchronization exchange is reported through the syncAgeMs diagnostic; however, the implemented firmware does not impose a separate model-expiration threshold based solely on this age. A node that stops providing valid data for more than 3 s is marked inactive, and detected node-clock discontinuities cause the clock model to be cleared and rebuilt. When a valid mapping is unavailable, the data packet retains its original node-local timestamps and synchronization-invalid status, while the mapped master timestamp and conversion slope are set to zero. Such samples are retained in the recording rather than being silently discarded or represented as synchronized measurements.

### 2.7. Host Acquisition and Visualization Software

A custom host application, Multimodal Sensor Studio, was developed in Python (Python Software Foundation, Beaverton, OR, USA) using PySide6 (The Qt Company, Espoo, Finland), PyQtGraph (PyQtGraph developers), pyserial (Chris Liechti), NumPy (NumPy developers), and SciPy (SciPy developers) (Figure 4). The application receives the binary data and diagnostic frames transferred by the central gateway and provides real-time visualization, recording, and session-level control of the multimodal acquisition system.

The seven available data streams can be controlled independently at the host level. For each stream, the operator can separately choose whether it is displayed in real time and recorded to disk, allowing the visualization and recording configuration to be adapted to the requirements of each session. Recorded data are stored as CSV files while retaining the required source sample indices and timing information for synchronized analysis. The recording pipeline preserves the available per-sample timing information and source indices rather than replacing them with a uniform sampling interval derived from the nominal configuration. The synchronized master timestamps are retained for subsequent temporal alignment and analysis, and no uniform resampling is applied to the raw data during acquisition or CSV storage. Nevertheless, timestamp precision depends on the acquisition mechanism of each modality: in particular, timestamps for earlier samples within a multi-sample PPG FIFO read are reconstructed using the configured 2.5 ms interval rather than independently measuring each optical conversion instant. When a uniformly sampled signal is required for a specific offline analysis, such as the EMG common-input cross-correlation test, interpolation is performed explicitly using the recorded timing information. The stream-specific files retain the corresponding raw or calibrated measurements: red and infrared values for PPG, three accelerometer and three gyroscope axes for IMU, and raw counts, calibrated force, and battery information for force data.

Live sEMG display filtering can be applied for visualization without modifying the raw EMG data stored to disk. Likewise, force calibration and zeroing are applied at the host level while retaining the underlying HX711 measurements. The application additionally supports participant/session identifiers, session-folder generation, force tare/auto-zero, event markers, synchronization diagnostics, and transport diagnostics. The representative application screenshot was captured during an informal author-performed functionality check. The displayed signals were used exclusively to illustrate simultaneous visualization and were not considered part of the experimental validation results.

### 2.8. Experimental Validation

Validation focused on the system-level properties introduced by the multimodal wireless architecture rather than re-characterizing the metrological performance of the commercial PPG/IMU sensors or repeating the previously reported transducer-specific validation of the force devices [14]. Six validation protocols were considered: (1) full-system sustained operation, (2) external EMG2–EMG3 common-input timing validation, (3) full-network GPIO reference-pulse clock-alignment validation, (4) clock-model parameter sensitivity analysis, (5) wireless network-load testing, and (6) distance and obstruction testing. Battery operating duration was additionally characterized under continuous sensor acquisition and wireless streaming conditions. This separation allowed acquisition stability, packet continuity, synchronization accuracy, wireless loading, and propagation conditions to be evaluated independently [6,7,8,11].

#### 2.8.1. Full-System Multi-Rate Sustained-Operation Test

All six physical wireless nodes and all seven data streams were operated simultaneously under maximum normal system load. The nodes were positioned approximately 1 m from the central gateway under line-of-sight laboratory conditions, and all the streams were enabled for recording. Three independent 30-min recordings were acquired.

For each recorded stream, the effective recorded sampling frequency and source-index cadence were calculated using the synchronized master time interval spanning the first and last recorded samples:

(9) $$f_{\mathrm{eff}}=\frac{\left(N_{\mathrm{received}}-1\right)\times 10^{6}}{t_{\mathrm{last}}^{M}-t_{\mathrm{first}}^{M}},\;f_{\mathrm{index}}=\frac{\left(i_{\mathrm{last}}-i_{\mathrm{first}}\right)\times 10^{6}}{t_{\mathrm{last}}^{M}-t_{\mathrm{first}}^{M}}.$$

Here, $t^{M}$ denotes the sample timestamp mapped to the gateway master-clock domain in microseconds, $N_{\mathrm{received}}$ is the number of recorded samples, and *i* is the source-generated sample index. The effective recorded rate, $f_{\mathrm{eff}}$, includes the effect of missing samples, whereas $f_{\mathrm{index}}$ estimates the source-index progression rate over the same observation interval. Comparing these quantities separates the contribution of source-index gaps from the remaining difference relative to the configured nominal sampling rate. However, the index-based rate does not independently measure the physical sensor oscillator frequency or identify samples lost before source-index assignment. Sample loss was expressed as a percentage according to

(10) $$Loss_{sample}\left(\%\right)=\left(1-\frac{N_{received}}{i_{max}-i_{min}+1}\right)\times 100.$$

The corresponding sample-continuity percentage was calculated as $100-Loss_{sample}$. In addition, packet-sequence gaps, sample-index gaps, duplicate and out-of-order packets, source-error counters, source queue-drop counters, gateway receive-queue drops, USB queue drops, USB sequence gaps, CRC errors, synchronization-valid fraction, synchronization-fit RMS, RTT, and estimated clock drift in ppm were retained as diagnostic outcomes. Synchronization-fit RMS is reported as the residual of the node-to-master clock model and is not interpreted as an independent measure of absolute inter-node synchronization accuracy. Synchronization-map coverage was calculated as the percentage of retained sensor samples whose received data packets carried a valid node-to-master mapping flag, $C_{\mathrm{sync}}=100N_{\mathrm{mapped}}/N_{\mathrm{recorded}}$, where $N_{\mathrm{mapped}}$ is the number of recorded samples marked as synchronization-valid and $N_{\mathrm{recorded}}$ is the total number of recorded samples. This metric describes the availability of an internally accepted clock mapping for the retained data; it does not include missing or undelivered samples and does not quantify absolute timing accuracy, mapping freshness, or the uncertainty of extrapolated clock estimates. Effective recorded rates were summarized across the three repetitions using mean and standard deviation, whereas packet and sample continuity were calculated as pooled percentages over the corresponding recordings. As a supplementary data-integrity check, consecutive node-local and master-mapped timestamps were tested for negative differences in source-index order separately from missing sample indices and the firmware-defined synchronization-valid flag.

#### 2.8.2. External Inter-Node Synchronization Validation

An independent common electrical signal was acquired simultaneously by two physically independent sEMG nodes (EMG2 and EMG3) to quantify end-to-end inter-node temporal alignment. EMG2 and EMG3 were selected as a representative pair of physically independent but hardware-identical 2 kHz nodes, allowing inter-node clock alignment to be evaluated without introducing differences in sensing hardware or sampling configuration. A laboratory signal generator produced a 1 Hz periodic pulse. Its output was split directly to the two sEMG inputs, which shared a common reference/ground, and the signal level was adjusted to remain within the non-saturated operating range of both complete front ends. Three independent 5-min recordings were acquired. Only EMG2 and EMG3 were active during this test, positioned approximately 1 m from the gateway under laboratory line-of-sight conditions. Each 2 kHz stream transmitted 20 samples per packet, yielding approximately 200 sensor-data packets/s in aggregate excluding synchronization messages. This experiment therefore provides an external validation of relative timing alignment for the tested EMG2–EMG3 node pair while not independently quantifying modality-specific acquisition delays in the PPG, IMU, or force sensing interfaces.

Pulse candidates were detected from the EMG2 waveform using the absolute deviation from its local median together with a refractory interval matched to the pulse period. For every detected event, a 0.5 s window from EMG2 and EMG3 was interpolated onto a common 2 kHz time grid. The relative lag was estimated from the peak absolute cross-correlation between the two windows followed by parabolic sub-sample refinement around the correlation maximum. Windows with an absolute waveform-correlation coefficient below 0.60 were excluded. For accepted event *k*, the corrected timing error was defined as

(11) $$e_{k}=t_{EMG3,k}^{master}-t_{EMG2,k}^{master}.$$

An offline initial-offset-only comparison was additionally constructed from the node-local timestamps by applying the initial node-to-master offset estimated during the first 5 s of each recording while omitting clock-rate correction. Bias, standard deviation, MAE, RMSE, 95th-percentile absolute error, maximum absolute error, and the linear timing-error slope versus elapsed time were calculated for both alignment methods. To account for between-recording variability, timing-performance metrics were calculated separately for each of the three experimental recordings. The signed mean timing difference was reported as temporal skew (bias) together with MAE, RMSE, the 95th-percentile and maximum absolute timing errors, and the linear timing-error trend. The individual recordings, rather than the 897 accepted pulse events, were treated as the independent experimental units for statistical summaries. In addition to pooled descriptive statistics, 95% confidence intervals for the mean run-level MAE and RMSE were calculated using Student’s *t* distribution with two DoFs. The associated synchronization diagnostics, including RTT, estimated clock drift, fit RMS, and accepted fit-point count, were logged approximately once per second for both EMG nodes during all three recordings.

#### 2.8.3. Full-Network GPIO Reference-Pulse Clock-Alignment Validation

To independently assess node-level clock alignment under heterogeneous sensing workloads, a common 1 Hz digital reference pulse was distributed to dedicated GPIO inputs on all six ESP32-C3 sensor nodes and the ESP32-S3 gateway. Each node captured the rising-edge time on its local microsecond timer and transmitted a reference-event record separately from its sensor-data packets. The gateway captured the same pulse in its own clock domain and converted node-event timestamps using the node-to-master clock model. For each node, reference events were matched in increasing order to the nearest unused gateway edge, using the available node-to-master mapping and a 250 ms maximum matching tolerance. The timestamp mapping used the available gateway clock model when the reference packet was processed; consequently, changes in the model between edge capture and packet processing are part of the measured procedure. A valid comparison required a valid clock model and an associated gateway reference event. The signed event error was calculated as

(12) $$e_{i,k}^{\mathrm{GPIO}}={\hat{t}}_{i,k}^{\mathrm{master}}-t_{\mathrm{gateway},k},$$

where *i* indexes the six wireless nodes and *k* indexes corresponding reference events. Three approximately five-minute recording intervals were obtained while the nodes and gateway remained powered and the clocks continued running. The accompanying raw gateway USB recordings confirmed that all seven sensor streams were concurrently active during each interval. The GPIO-level test bypassed sensor front-ends; hence, it characterizes node-to-master clock mapping and GPIO event timestamping, not the conversion, filtering, FIFO, or polling latency of the sensing elements. Separate recording intervals are not interpreted as independent power-cycle replications.

As a complementary cross-modality analysis, GPIO reference events from two different physical nodes were paired only if both events had valid clock mappings, belonged to the same recording interval, and were matched to the same gateway reference pulse. For nodes *i* and *j*, the signed pairwise alignment error was calculated by subtracting their node-to-gateway GPIO errors, thereby cancelling the common gateway reference timestamp:

(13) $$e_{i,j,k}^{\mathrm{pair}}=e_{i,k}^{\mathrm{GPIO}}-e_{j,k}^{\mathrm{GPIO}}={\hat{t}}_{i,k}^{\mathrm{master}}-{\hat{t}}_{j,k}^{\mathrm{master}}.$$

Pairwise signed bias, MAE, RMSE, 95th-percentile absolute error (P95), and the fraction of paired events with absolute error no greater than 1 ms were evaluated for all 11 pairs connecting different sensing modalities or node types. Because the same devices and reference pulses contribute to multiple pairwise comparisons, pooled event-level statistics were treated as descriptive measurements rather than independent hardware replications.

#### 2.8.4. Clock-Model Parameter Sensitivity Analysis

A separate 5-min experiment was conducted under full-system operating conditions with all six wireless nodes and seven sensor streams active at approximately 1 m line of sight. Individual four-timestamp synchronization exchanges were recorded while retaining the operational clock-model parameters.

The recorded exchanges were subsequently reprocessed offline using nine parameter configurations: rolling-window sizes of 16, 32, and 64 observations crossed with RTT acceptance margins of 1.5, 3.0, and 5.0 ms. For each configuration, an independent rolling history with the specified maximum length was used. Only exchanges satisfying the absolute 10 ms RTT ceiling entered the history, and at least four observations retained by the relative RTT filter were required for a valid regression fit. To make the nine configurations directly comparable, the first 64 accepted exchanges of each node were excluded from all the reported steady-state summaries, leaving 951 evaluation opportunities across the six nodes. Valid-fit counts were summed across nodes; fit-RMS residuals, numbers of fitted observations, and absolute differences between successive valid drift estimates were first summarized by their median within each node and then by the median across nodes. The internal fit statistics quantify estimator consistency, not independent timing accuracy. In particular, zero median drift-update variation may arise when a restrictive RTT filter repeatedly selects an unchanged set of observations and should not be interpreted as superior clock tracking.

#### 2.8.5. Wireless Network-Load Test

Wireless network loading was evaluated at approximately 1 m line of sight using three discrete system configurations: (1) one active EMG node, (2) all three EMG nodes active simultaneously, and (3) the complete six-node/seven-stream system. No intermediate two-EMG condition was tested. In the reduced-load conditions, only the stated EMG node or nodes were active; the PPG/IMU and force nodes were not active. The one-EMG and three-EMG conditions were each recorded for 5 min and repeated three times. The full-system condition was represented by the three 30-min sustained-operation recordings acquired under the same approximately 1 m line-of-sight geometry, providing a longer observation period for the maximum-load condition without repeating an equivalent acquisition.

Packet delivery ratio (PDR) was calculated from source packet sequence numbers as

(14) $$PDR=\frac{N_{received}}{N_{received}+N_{missing}}\times 100\%.$$

Because source packet sequence numbers are assigned when packets are generated, the reported PDR represents end-to-end source-to-gateway packet delivery. Sequence gaps may therefore reflect either node-side transmit-queue drops or packets not successfully delivered over the wireless link; the metric should not be interpreted as a pure over-the-air packet error rate.

To further characterize packet-loss mechanisms, source-side packet-discard counters were examined alongside packet-sequence gaps. For the EMG nodes, these counters include packets discarded because of transmission-queue saturation and packets intentionally discarded during link-down handling. Gateway receive-queue drops, USB queue drops, CRC errors, and USB sequence discontinuities were evaluated separately to distinguish upstream transmission-related losses from downstream data-transport failures. Because PPG and IMU share a physical transmission node and its diagnostic counters, shared counters were counted only once when calculating node-level discard totals.

In addition, packet and sample loss, synchronization-valid fraction, synchronization-fit RMS, RTT, gateway queue drops, USB queue drops, USB sequence gaps, and CRC failures were evaluated. For the single-EMG condition, synchronization statistics were calculated only for the active node.

#### 2.8.6. Wireless Distance and Obstruction Test

Wireless distance testing was performed with all six physical sensor nodes and all seven data streams active simultaneously. The approximately 1 m line-of-sight reference condition was provided by the three full-system sustained-operation recordings described above. Additional line-of-sight measurements were then acquired at approximately 5 m and 10 m with three independent 5-min recordings at each distance. Finally, an obstructed non-line-of-sight condition was evaluated at approximately 5 m using three additional 5-min recordings. In this condition, the sensor nodes and central gateway were located in separate rooms with a 20-cm-thick concrete wall and a closed fire-protection door along the propagation path. The same packet-continuity and synchronization metrics used in the network-load experiment were calculated. The line-of-sight conditions were selected to represent the intended short-range laboratory use of the platform, with approximately 1–6 m considered the primary operating range and 10 m evaluated as an extended-range condition. The obstructed condition was included as a robustness test rather than a recommended operating configuration. All the distance and obstruction tests were conducted in an indoor laboratory environment using ESP-NOW on a fixed 2.4 GHz wireless channel (channel 1) with all six physical sensor nodes and seven data streams active. The LOS measurements were performed at nominal node-to-gateway separations of 1, 5, and 10 m. For the 5 m NLOS condition, the nodes and gateway were positioned in separate rooms with a 20-cm-thick concrete wall and a closed fire-protection door along the propagation path. The experiments were performed as separate recording conditions rather than a controlled radio-propagation characterization. Individual antenna orientations, received signal strength (RSSI), and background wireless channel occupancy were not systematically characterized, and a randomized or counterbalanced test order was not documented. Therefore, the measurements are interpreted as configuration-specific communication-performance observations rather than an isolated assessment of obstacle attenuation.

#### 2.8.7. Battery Operating-Duration Test

Battery operating duration was evaluated separately from the full-system sustained-operation tests. The sensor nodes were operated with fully charged batteries under continuous acquisition and wireless data-streaming conditions. The experimentally observed operating durations were recorded for the evaluated sensor-node configurations.

## 3. Results

### 3.1. Full-System Sampling and Data-Integrity Performance

The three simultaneous full-system recordings lasted 1800.687, 1800.485, and 1800.593 s. The corresponding run-level packet delivery ratios were 100.000%, 99.988%, and 99.974%, yielding a pooled PDR of 99.987% across the three recordings. The pooled sample-continuity rate was 99.988%. All the retained samples in the seven data streams were marked with a valid node-to-master clock mapping corresponding to 100% synchronization-map coverage during the recorded intervals. This value indicates that an internally accepted regression model was available when the packets were processed; it does not represent an independent verification of absolute synchronization accuracy. No gateway receive-queue drops, USB queue drops, CRC errors, USB sequence gaps, duplicate packets, or out-of-order packets were observed during the sustained-operation recordings.

Table 4 distinguishes the configured nominal acquisition rates, source-index cadence, and effective recorded sampling rates calculated from synchronized master timestamps. The difference between source-index cadence and effective recorded rate reflects sample-index gaps in the retained data. For example, EMG2 exhibited a source-index cadence of 1999.912 samples/s but an effective recorded rate of 1999.686 samples/s, consistent with the observed missing samples. Similarly, PPG and IMU exhibited source-index cadences of 398.674 and 414.765 samples/s, respectively, compared with effective recorded rates of 398.434 and 414.506 samples/s. Their source-index cadences were also slightly below the configured sensor rates, indicating that missing recorded samples alone do not explain the entire difference. The remaining discrepancy may arise from sensor-clock characteristics, sensor-data availability, or firmware acquisition behavior; these contributions cannot be uniquely separated without an independent sensor-clock reference. In contrast, the pinch and grip streams exhibited no source-index gaps with observed acquisition cadences of 85.139 and 85.926 samples/s, respectively. These rates reflect the observed HX711 data-ready acquisition cadence under the implemented hardware configuration rather than software oversampling or an assumed fixed 80 Hz sampling interval.

Internal clock-model quality was consistent across the six wireless nodes during the sustained full-system recordings. Across the six wireless nodes, the mean internal synchronization-fit RMS ranged from 0.531 ms for the combined PPG/IMU node (Node 2) to 0.590 ms for the EMG3 node (Node 6), and mean RTT ranged from 6.05 to 6.22 ms. The mean estimated clock-rate offsets were node-specific: −48.8, −19.3, −43.6, −37.7, −44.2, and −43.9 ppm for EMG1, PPG/IMU, pinch, grip, EMG2, and EMG3, respectively. These fit residuals quantify the stability of the continuously updated node-to-master clock models across all six physical nodes. Independent external timing accuracy is evaluated separately below for the tested EMG2–EMG3 pair using the common-input experiment.

An additional timestamp-order analysis of the three 30-min full-system recordings confirmed that timestamp backsteps were infrequent and did not coincide with source-index discontinuities. For PPG, 242 intervals (0.0112% of retained adjacent intervals) exhibited backsteps in both node-local and master-mapped time; their median, 95th-percentile, and maximum magnitudes were 0.500, 1.500, and 1.546 ms, respectively, each below the nominal 2.5 ms PPG sampling interval. Across EMG1–EMG3, 72 master-mapped intervals (0.00022%) exhibited backsteps with a median magnitude of 0.0875 ms, 95th percentile of 0.4716 ms, and maximum of 0.710 ms. Node-local sEMG timestamps remained increasing, all 72 events occurred at the boundaries of 20-sample packets, and 70 of the 72 backsteps were smaller than the nominal 0.5 ms sEMG sampling interval. All 314 PPG and sEMG events retained consecutive source indices and valid clock-mapping flags; no corresponding non-monotonic intervals were identified in the retained IMU or force streams. These rare timestamp-order anomalies are distinct from sample loss and the firmware-defined synchronization-map coverage metric.

### 3.2. External EMG2–EMG3 Inter-Node Synchronization Accuracy

Across the three 5-min common-input recordings, 299, 299, and 300 pulse events were detected. A total of 897 of 898 detected events (99.89%) satisfied the waveform-correlation acceptance criterion.

Table 5 presents the synchronization results separately for the three recordings and as pooled event-level summaries. Drift compensation reduced the MAE in all three recordings, from 0.3427 to 0.0761 ms in Run 1, from 0.1060 to 0.0733 ms in Run 2, and from 0.0863 to 0.0767 ms in Run 3. The corrected run-level MAEs were closely grouped between 0.0733 and 0.0767 ms, with a mean of 0.0754 ms and a 95% confidence interval of 0.0708–0.0799 ms. The mean run-level RMSE was 0.1269 ms (95% confidence interval: 0.0845–0.1694 ms). The absolute magnitude of the timing-error trend also decreased in every recording. For the pooled 897 accepted events, drift compensation reduced MAE from 0.1780 to 0.0754 ms, RMSE from 0.2373 to 0.1277 ms, and the 95th-percentile absolute error from 0.5079 to 0.2781 ms. The number of events with an absolute timing error exceeding 0.5 ms decreased from 57 with initial-offset-only alignment to 15 after drift compensation. However, the maximum absolute error increased from 0.5670 to 0.6879 ms, indicating that the reduction in typical error and systematic drift did not eliminate all the individual timing excursions. Figure 5 illustrates the temporal error distribution and the overall effect of drift compensation across the recordings.

An additional examination of the raw recordings and synchronization diagnostics showed that the localized excursions in Figure 5a coincided with transient variations in the estimated clock mappings. The recorded sample sequences remained continuous, and the waveform-correlation quality remained high.

### 3.3. Full-Network GPIO Reference-Pulse Clock-Alignment Accuracy

Across three approximately five-minute recording intervals, 5321 node reference-event records were obtained, of which 5319 had a valid clock mapping and gateway-event match. All six nodes contributed valid events during every interval. The pooled absolute timing error was characterized by an MAE of 0.3123 ms, RMSE of 0.3822 ms, and 95th percentile of 0.7272 ms. A total of 5292/5319 accepted events (99.49%) had absolute errors no greater than 1 ms; the maximum absolute error was 1.260 ms. The recording-level summaries are reported in Table 6 and the per-node summaries in Table 7. These values quantify the combined external GPIO event timestamping and node-to-master clock-alignment error, not sensor-sample acquisition latency.

A complementary analysis quantified relative GPIO timing error directly between heterogeneous wireless nodes by pairing reference events associated with the same gateway pulse. As summarized in Table 8, the 11 cross-modality physical-node pairs each contributed 865–890 matched reference pulses across the three recording intervals. Pairwise MAE ranged from 0.3158 to 0.4983 ms, and P95 absolute error ranged from 0.7970 to 1.1436 ms. The proportion of events with absolute pairwise errors no greater than 1 ms ranged from 91.80% to 98.62%. For example, the EMG2–PPG/IMU, EMG2–pinch, and EMG2–grip pairs yielded MAEs of 0.3468, 0.3459, and 0.3285 ms, respectively. Four of the 11 pairs had P95 errors above 1 ms, and the largest individual pairwise absolute error was 2.054 ms. These results provide external evidence of relative GPIO-level clock alignment between nodes with different sensing functions under full acquisition load without implying equivalent timing accuracy for physical events within the modality-specific sensor acquisition chains.

### 3.4. Clock-Model Parameter Sensitivity Analysis

The separate five-minute full-system recording contained 1885 four-timestamp synchronization exchanges, of which 1335 met the absolute 10 ms RTT ceiling; no USB sequence gaps or CRC failures were detected. The median RTT among accepted exchanges was 6.074 ms, and the median node-side processing interval was 0.071 ms. Table 9 reports all nine offline configurations using a common steady-state evaluation interval following 64 accepted exchanges per node (951 opportunities across six nodes). At a 32-observation window, tightening the RTT margin from 3.0 to 1.5 ms reduced valid-fit availability from 951/951 to 863/951 and reduced the median internal fit RMS from 0.600 to 0.246 ms. Relaxing the margin to 5.0 ms retained 951/951 valid fits but increased the median internal RMS to 0.872 ms. At a 3.0 ms RTT margin, increasing the window length from 16 to 32 and 64 observations reduced the median change between successive valid drift estimates from 12.08 to 2.72 and 0.58 ppm, while the median internal fit RMS values were 0.540, 0.600, and 0.611 ms, respectively. At the 3.0 ms RTT margin, the 64-observation configuration retained a median of 33.5 observations per fit, confirming that the offline replay used more than the 32-observation operational history. The 32-observation and 3.0 ms configuration was retained as an engineering trade-off between temporal responsiveness, fit availability, and estimator stability rather than a universally optimal setting. Results with zero median drift-update changes under the strictest filtering settings may reflect an unchanged fitted subset rather than high tracking accuracy.

### 3.5. Wireless Network-Load Performance

No packet loss, sample loss, source queue drops, gateway receive-queue drops, USB queue drops, CRC errors, or USB sequence gaps were observed in any of the three repetitions of either the one-EMG or three-EMG condition. Under the complete six-node/seven-stream condition, the pooled PDR decreased slightly to 99.987% and sample loss increased to 0.012%. No gateway receive-queue drops, USB queue drops, CRC errors, or USB sequence gaps were observed, indicating that the small amount of data loss occurred upstream of the gateway-to-host transport. The mean synchronization RTT increased with active network load, while all the recorded samples carried valid node-to-master mappings under the firmware-defined criterion. The network-load results are summarized in Table 10.

### 3.6. Wireless Distance and Obstruction Performance

Wireless distance testing demonstrated high packet-delivery reliability across the intended short-range operating conditions. Under full six-node/seven-stream operation, pooled PDR reached 99.987% at approximately 1 m LOS and 99.913% at 5 m LOS while remaining at 96.398% under the extended 10 m LOS condition. At 10 m, the three EMG nodes exhibited distinct delivery characteristics despite using the same acquisition and transmission architecture: EMG1 and EMG3 achieved pooled PDRs of 94.625% and 91.449%, respectively, whereas EMG2 retained 100.000% packet delivery. The PPG, IMU, and force streams each maintained a PDR of at least 99.87%.

Additional analysis of source-side diagnostics showed that EMG1 and EMG3 accounted for 12,566 of the 12,636 missing packets across the three 10-m recordings (99.45%). These two nodes also reported 12,209 source-side packet-discard events, including transmission-queue and link-down-related discards. No gateway receive-queue drops, USB queue drops, CRC errors, or USB frame-sequence gaps were detected. These findings indicate that the observed degradation was concentrated in specific upstream transmission paths rather than the shared gateway-to-host transport. Although source-side discards account for a substantial portion of the observed missing-packet events, the aggregate diagnostics do not uniquely separate every local discard from an unsuccessful wireless delivery.

The 5 m NLOS condition, with the central gateway and sensor nodes located in separate rooms behind a 20-cm-thick concrete wall and a closed fire-protection door, yielded a pooled PDR of 99.187%. The synchronization-valid fraction remained 100% in all nine 5 m/10 m distance recordings. Across the distance and obstruction tests, no gateway receive-queue or USB queue drops occurred. One 5 m LOS recording contained two CRC errors and five USB sequence gaps; the other eight distance/obstruction recordings contained none. Table 11 summarizes the distance and obstruction results. The three 5 m LOS recordings yielded run-level PDRs of 99.996%, 100.000%, and 99.744%, whereas the three 5 m NLOS recordings yielded 98.756%, 99.400%, and 99.405%. The 5 m NLOS condition exhibited 2849 missing source packets and 3197 source transmit-queue-drop counter increments compared with 304 missing packets and 290 queue-drop counter increments in the 5 m LOS condition. These counts describe different diagnostic events and should not be subtracted directly to infer over-the-air packet loss.

### 3.7. Battery Operating Duration

Experimental battery-autonomy measurements under continuous acquisition and wireless streaming demonstrated approximately 2 h of operation for the EMG nodes, 3 h for the combined PPG/IMU node, and 6.15 h for the evaluated force-node configuration. These measured operating durations demonstrate the feasibility of battery-powered operation during the intended short-duration multimodal acquisition sessions.

## 4. Discussion

The principal contribution of this study is an integrated experimentally evaluated architecture for heterogeneous multi-rate acquisition rather than a new individual clock-synchronization algorithm. Six physical wireless nodes produce seven physiological and biomechanical streams, retain modality-specific acquisition rates and source measurements, and share a single ESP-NOW network for sensor data and clock-synchronization exchanges. The gateway maintains per-node clock mappings alongside packet-, sample-, synchronization-, and host-transport diagnostics. Three sustained full-system recordings, external common-input and six-node reference-pulse validation, and network-load and distance tests collectively demonstrate the utility of this coordinated design for short-range laboratory measurement. This combination supports simultaneous acquisition without imposing a common sampling frequency on different sensors.

The external EMG2–EMG3 experiment provides direct evidence of the relative timing performance achieved between two physically independent high-rate nodes. The pooled MAE of 0.075 ms and 95th-percentile absolute error of 0.278 ms were both below the 0.5 ms sampling interval of the 2 kHz sEMG signals. More importantly, the reduction in the systematic timing-error trend from 0.074 ms/min with initial-offset-only alignment to 0.005 ms/min after drift compensation demonstrates the practical value of continuously estimating the node-to-master clock-rate relationship rather than applying only an initial offset correction. The near-zero pooled bias should not be interpreted as microsecond-level absolute timing accuracy because the external timing estimates were obtained using cross-correlation and sub-sample interpolation. The 100% synchronization-map coverage reported for the retained samples denotes the availability of an internally accepted node-to-master regression mapping. During temporarily delayed or rejected synchronization responses, the last valid mapping may remain in use, so this metric is not an independent absolute-timing-accuracy or model-freshness criterion. The external reference-event tests provide separate evidence of temporal alignment.

The recording-level analysis provides additional evidence of repeatable synchronization performance within the tested device pair: the drift-compensated MAE remained between 0.0733 and 0.0767 ms across the three independent recording sessions, and the magnitude of the estimated timing-error trend decreased in every session. Although some individual maximum errors and run-specific higher-percentile metrics increased after compensation, the reduction in pooled error and the number of events exceeding 0.5 ms indicate improved overall temporal alignment. The confidence intervals were calculated across the three recordings rather than treating individual pulse events as independent experimental replicates; consequently, they describe between-session variability for the tested hardware pair rather than generalization across different devices or experimental environments.

The additional six-node external GPIO-pulse test assesses a different quantity: relative node-to-master clock alignment for heterogeneous wireless devices under simultaneous acquisition load. Its pooled MAE (0.3123 ms) and P95 (0.7272 ms) support sub-millisecond typical clock-alignment performance under the tested conditions, but the observed 1.260 ms maximum excludes a universal sub-millisecond bound. Neither this GPIO test nor the identical-EMG common-input test establishes sub-millisecond physical-event timing for the PPG, IMU, and force acquisition chains, whose internal conversion, filtering, and polling latencies require separate characterization. Prior synchronized wireless systems have also reported high timing precision using different communication and synchronization architectures [7,8,9]. However, direct numerical comparison is limited by differences in sampling rates, timestamp placement, radio protocols, oscillator characteristics, and validation methods. Compared with synchronization-focused systems, the present platform emphasizes not only inter-node timing accuracy but also sustained heterogeneous acquisition across multiple physiological and biomechanical modalities, synchronization exchanges within the same wireless network, modality-specific packet structures, and end-to-end integrity diagnostics across the source-to-host acquisition path.

The matched-pulse pairwise analysis extends the six-node GPIO evaluation by directly comparing the clock mappings of physically separate devices with different sensing functions. Across 11 heterogeneous pairs operating under the complete seven-stream load, the pairwise MAEs were 0.3158–0.4983 ms, and 91.80–98.62% of the matched events exhibited absolute errors no greater than 1 ms. The observed P95 values above 1 ms for four pairs and occasional larger individual errors preclude a universal sub-millisecond inter-node bound. Moreover, the common GPIO inputs bypass the optical, inertial, and force conversion pathways; the pairwise results strengthen the evidence for heterogeneous node-clock alignment but cannot establish acquisition-timing accuracy across the corresponding sensing modalities. The sensitivity analysis further demonstrates that the synchronization estimator remains dependent on the balance between RTT-based observation selection and regression-window length. A stricter RTT threshold reduces the contribution of higher-delay observations but may also decrease the availability of valid regression estimates, whereas longer regression windows provide smoother clock-rate estimates. Importantly, RTT filtering cannot guarantee symmetric forward and reverse communication delays when timestamps are captured at software-level interfaces. The internal fit residuals therefore characterize clock-model consistency, while the external common-input and GPIO-reference experiments provide separate empirical evidence of temporal alignment under the evaluated operating conditions.

The timestamp-order analysis identified infrequent non-monotonic intervals in the PPG and sEMG streams without accompanying source-index gaps. These events are consistent with the respective FIFO timestamp-reconstruction and packet-level clock-mapping procedures. Their low occurrence rates support the overall continuity of the retained data while emphasizing the importance of verifying timestamp ordering in downstream analyses that require a strictly monotonic time base.

The network-load experiments indicate that increasing the number of active streams affected synchronization-message timing more noticeably than the observed sample-mapping coverage. The mean synchronization RTT increased as the network progressed from one active EMG node to the complete six-node/seven-stream configuration, but the synchronization-valid coverage remained unchanged and only a small reduction in packet delivery was observed under full-system operation. The source-side diagnostic counters provided additional insight into the observed packet losses. In the extended-range measurements, missing packets were strongly concentrated in two high-rate EMG nodes and accompanied by source-side packet discards, while the gateway and USB transport remained free of detected queue losses. This behavior highlights the effectiveness of the system’s end-to-end diagnostic architecture in identifying the affected communication stage. The implemented link-recovery mechanism also allows sensor acquisition to continue during temporary transmission interruptions, prioritizing recent measurements when communication is restored.

Communication distance had a more pronounced influence on packet delivery than the increase in active network load at approximately 1 m, although this comparison should be interpreted descriptively because the corresponding experiments differed in duration and traffic composition. Packet delivery decreased with increasing line-of-sight distance, particularly at 10 m and predominantly in the high-rate EMG links. The 1 m and 5 m LOS conditions therefore provide the most representative evidence for the intended short-range laboratory operation, whereas 10 m is considered to be an extended-range test condition. At 5 m, the LOS configuration achieved a higher pooled PDR (99.913%) than the NLOS configuration (99.187%). The NLOS recordings exhibited more missing source packets (2849 versus 304) and source-side packet-discard counter increments (3197 versus 290). These findings are consistent with the more challenging communication conditions of the tested obstructed configuration, although the contributions of obstacle attenuation, antenna positioning, multipath propagation, and background wireless traffic were not independently isolated. Importantly, even under the obstructed 5 m condition, the system maintained 99.187% packet delivery while simultaneously acquiring all seven data streams across six wireless nodes, demonstrating reliable communication performance within the evaluated indoor environment. The observed node-specific differences among otherwise equivalent EMG units further demonstrate the value of integrated source-level diagnostics for identifying communication-performance variations within the wireless network.

Beyond communication performance, a further advantage of the architecture for multimodal research is the preservation of raw sensor measurements together with source sample indices and synchronized timing information. This allows subsequent signal-processing, calibration, and feature-extraction methods to be modified without altering the original acquired measurements. The architecture also distinguishes physical sensing nodes from logical data streams; for example, PPG and IMU share one physical node and node-to-master clock model while retaining independent sensor-side acquisition schedules and packet structures. This separation is particularly useful in heterogeneous sensing systems because synchronization can be maintained at the physical-node level without requiring all the sensing modalities to share the same acquisition process or sampling rate.

The primary objective of the present validation was to assess the integrated system’s multi-rate acquisition, inter-node temporal alignment, and wireless data integrity. Accordingly, the reported synchronization metrics characterize clock alignment rather than absolute sample-to-host delivery latency, while the analog front-end design parameters describe the implemented signal-conditioning architecture rather than a separate metrological qualification of the EMG circuitry.

## 5. Conclusions

This study demonstrates a six-node wireless architecture combining seven heterogeneous physiological and biomechanical streams, continuous node-to-master clock-rate compensation, raw-measurement and sample-timing preservation, and integrated source-to-host integrity diagnostics within a single ESP-NOW network. Three independent 30-min recordings achieved 99.987% pooled packet delivery and 99.988% sample continuity under simultaneous operation. Independent common-input and six-node reference-event experiments provided complementary evidence of temporal alignment at the tested interfaces. A supplementary analysis of 11 heterogeneous node pairs yielded GPIO-level relative-alignment MAEs of 0.3158–0.4983 ms without establishing the acquisition latency of the individual sensing elements. The distinguishing contribution is therefore the coordinated implementation and experimental validation of these complementary capabilities for sustained short-range multi-rate laboratory acquisition.

## Figures and Tables

**Figure 1:**
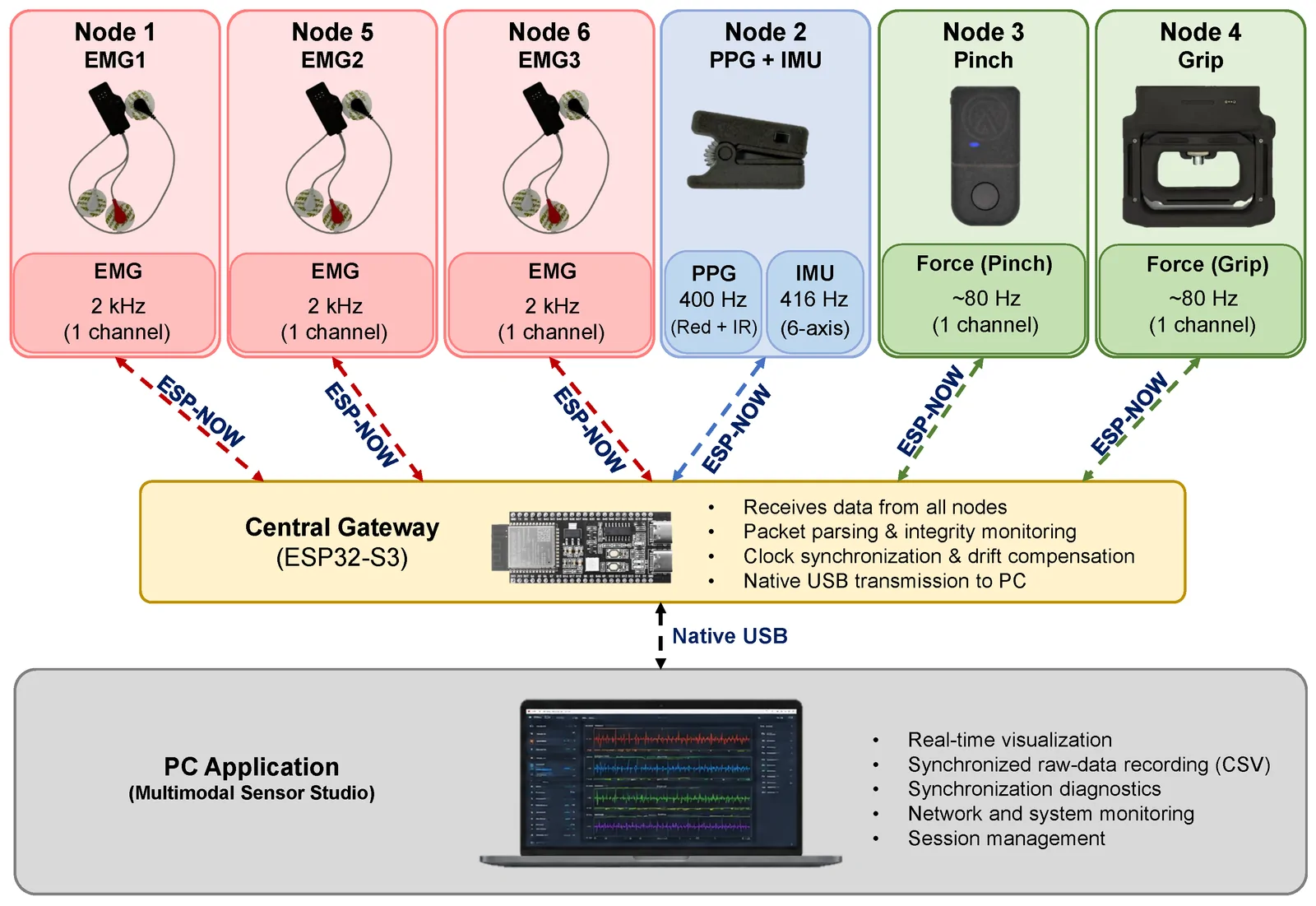
Overall architecture of the multimodal wireless acquisition system. The architecture comprises three independent sEMG nodes, one physical node combining PPG and IMU sensing, one pinch-force node, one grip-force node, an ESP32-S3 central gateway, and a host computer running the acquisition application.

**Figure 2:**
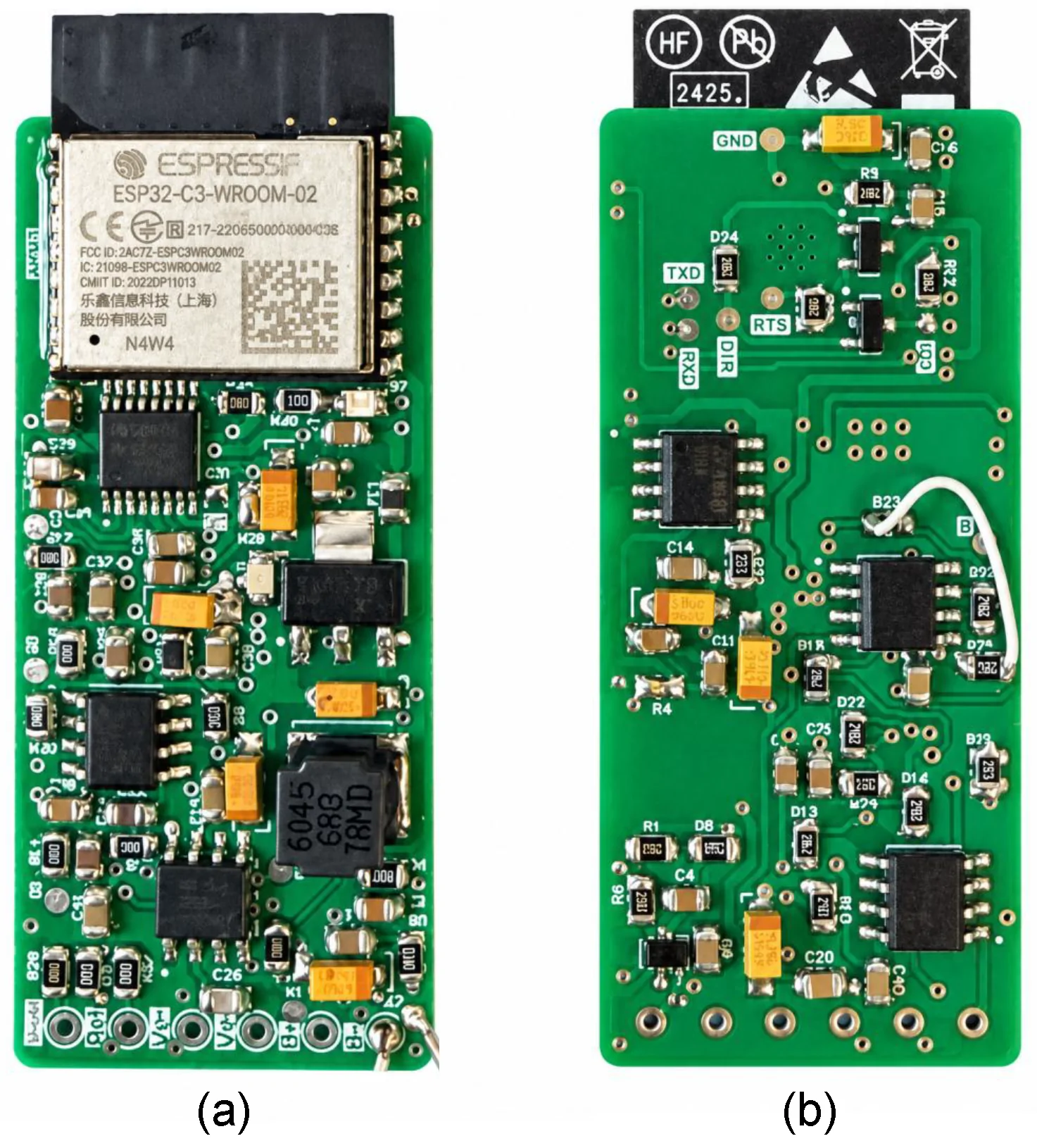
Implemented custom PCB of the wireless sEMG node used for EMG1, EMG2, and EMG3. (**a**) Top side and (**b**) bottom side.

**Figure 3:**
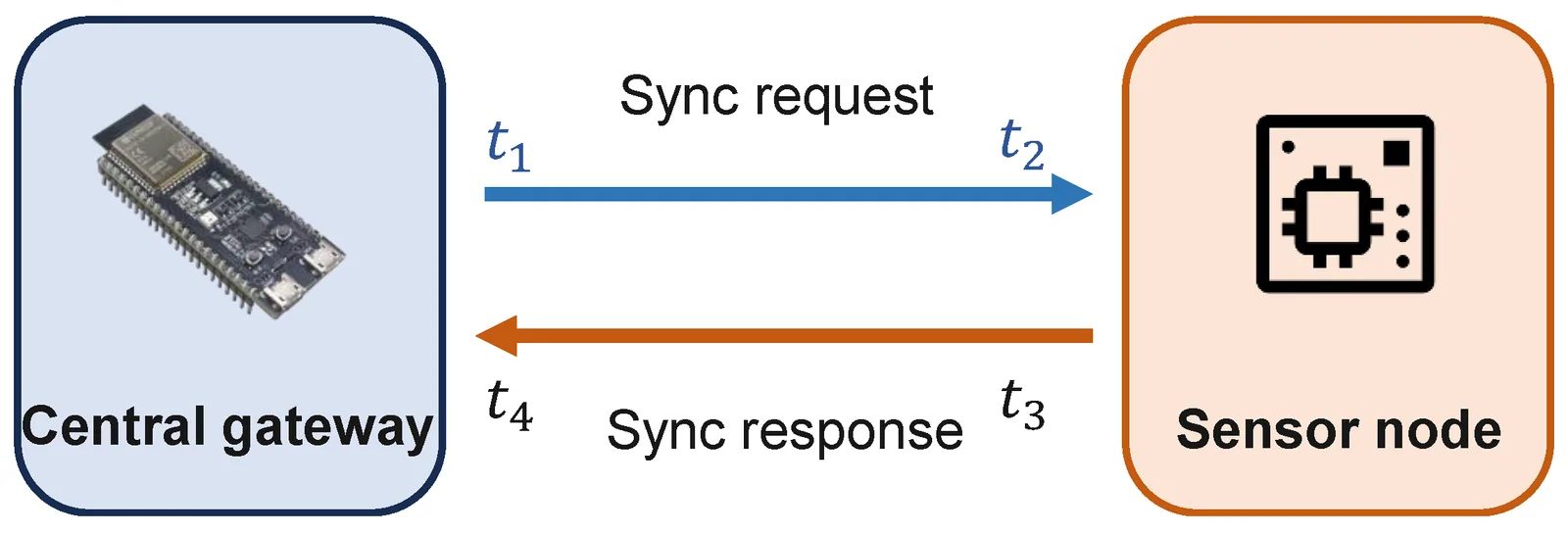
Two-way four-timestamp exchange used for wireless clock synchronization between the central gateway and an independently clocked sensor node. The gateway transmits the synchronization request at $t_{1}$, the node records reception and response transmission at $t_{2}$ and $t_{3}$, respectively, and the gateway records the returned response at $t_{4}$.

**Figure 4:**
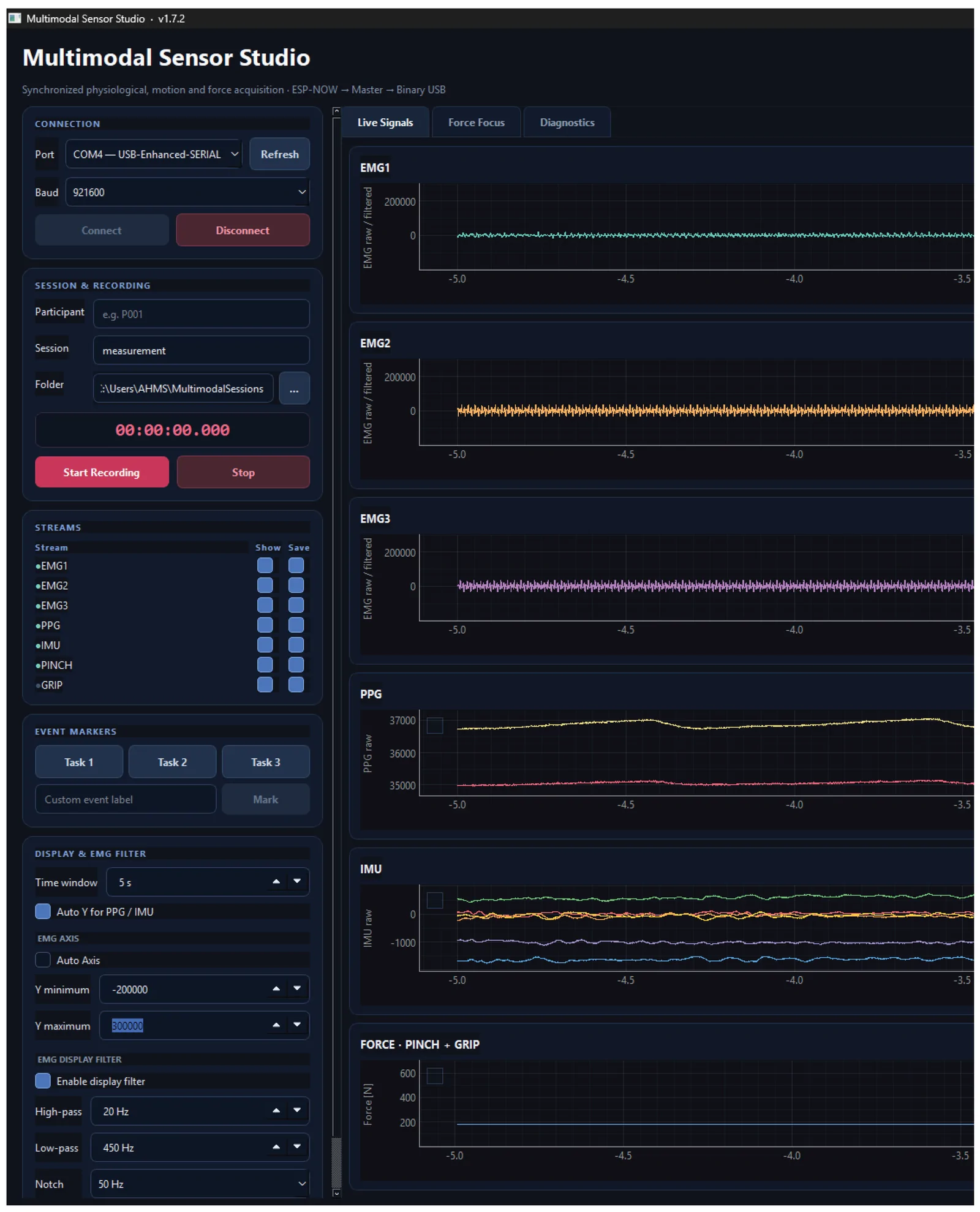
Multimodal Sensor Studio host application showing acquisition controls, per-stream recording and visualization settings, and real-time signal display. The displayed traces were obtained during informal self-testing by one of the authors solely to demonstrate the software interface and visualization functionality. Different colors are used to distinguish the displayed sensor channels and do not represent different experimental conditions or physiological classifications. They were not included in the quantitative validation datasets or used for physiological or clinical assessment.

**Figure 5:**
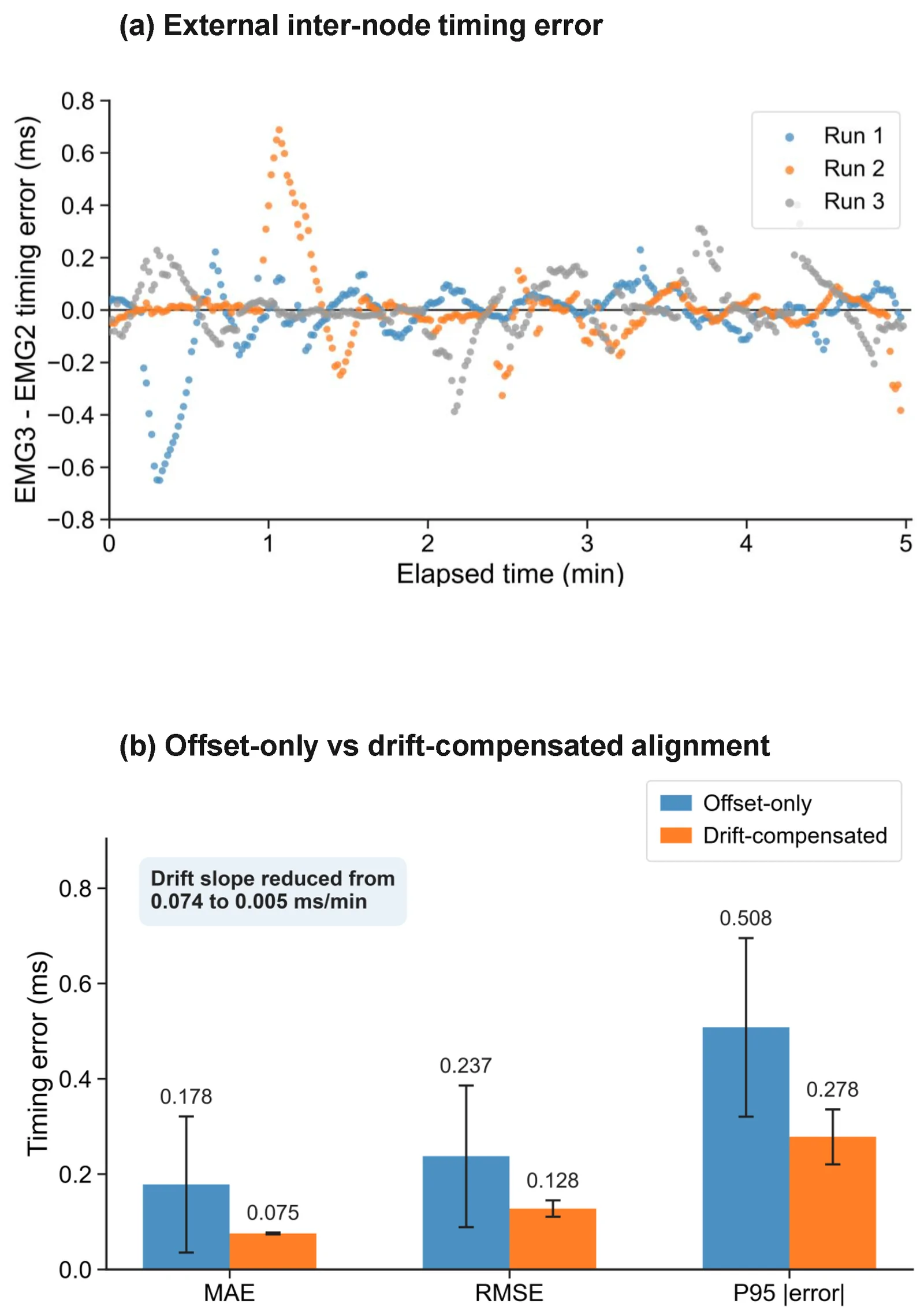
External inter-node synchronization validation using a common periodic electrical input simultaneously acquired by EMG2 and EMG3. (**a**) Inter-node timing error across three independent 5-min recordings after drift-compensated mapping to the common master time base. (**b**) Comparison of initial-offset-only and drift-compensated alignment in terms of mean absolute error (MAE), root mean square error (RMSE), and 95th-percentile absolute timing error. Error bars indicate the standard deviation across the three recordings.

**Table 1:** Technical comparison of representative wireless sensing and synchronization systems. Timing metrics are identified by their original measurement definitions and should not be interpreted as directly interchangeable.

Study	Nodes	Sensing Modalities	Sampling Rate	Wireless	Clock Synchronization	Verified Timing	Data Transmission	Power/Autonomy	Tested Range
Biagetti 2020 [5]	1 sensor module	EMG/ECG, IMU	Up to 3.2 kHz	BLE	Local acquisition timing; no separate inter-node accuracy metric	NR	BLE streaming and throughput assessed	NR	NR
Serrani 2024 [6]	2 sensors + gateway	Wearable biosignals	1 kHz (test)	Custom + BLE	Proprietary wireless clock coordination	1.18 clock ticks median inter-node synchronization delay; not external timing error	BLE throughput and loss diagnostics	NR	Up to 10 m
Sondej 2024 [7]	4 sensors + central	Generic synchronous measurements	Application-dependent	Proprietary 2.4 GHz	TDMA-based hardware-oriented synchronization	0.826 μs maximum sampling-start difference	Scheduled TDMA data transmission	15 μW per 1 kb/s throughput	0.5 m timing test
Biagetti 2025 [8]	1 sensor + receiver	sEMG	800 Hz (test)	BLE	Multi-clock-domain correction and dual-stage filtering	47 μs RMS timing jitter	Timestamped BLE packets	10–50% relative power saving	NR
Li 2023 [9]	2 sensors + central	Distributed biosignals	1 kHz	BLE	Application-layer timestamp pairing and linear regression	69 ± 71 μs (TI); 477 ± 490 μs (Nordic)	BLE notifications; latency target ≤ 30 ms	NR	NR
PhysioEdge 2025 [10]	2 nodes + timekeeper (sync test)	PCG, ECG, EMG, PPG, IMU	4–8 kHz audio; up to 3.2 kHz biopotential	Sub-1-GHz + Wi-Fi/BLE	Dedicated radio with synchronization-word interrupt	Hardware timing evaluated; comparable numeric statistic NR	Compressed multimodal data aggregation	4.9–25 mW in reported PCG communication tests	NR
MOT 2025 [11]	6 physical sensor nodes	sEMG	1 kHz	ESP-NOW	Gateway-side packet timestamping	Inter-node timing NR; latency < 3 ms reported	Packet loss below 0.5% in reported tests	72 min operating autonomy	70 m (6 ch); 100 m (2 ch)
Smelcerz 2026 [12]	Up to 5 nodes	Environmental/IoT telemetry	Approx. 1 Hz	ESP-NOW + MQTT	Three-probe two-way clock-offset estimation	Direct sensor alignment NR; uncertainty interval reported	5-node stored PDR approximately 96.7–97.0%	NR	NR
**This work**	**6 physical nodes**	**3 sEMG, PPG, IMU, pinch, grip; 7 streams**	**80 SPS nominal −2 kHz**	**ESP-NOW**	**Four-timestamp exchange; continuous per-node clock-drift compensation**	**75.4 μs MAE (EMG pair); 312.3 μs MAE (6-node GPIO)**	**99.987% PDR (3 × 30 min, 1 m LOS); packet/sample/USB diagnostics**	**2 h EMG; 3 h PPG/IMU; 6.15 h force (measured autonomy)**	**10 m LOS; 5 m NLOS**

Notes: NR denotes a quantitative measure that is not reported or directly comparable in the consulted source. Sampling rates and power figures refer to the specific configurations evaluated in each study. The 0.0754 ms value for this work represents common-input inter-EMG temporal alignment, whereas the 0.3123 ms value represents GPIO-level node-to-master clock alignment; neither represents a validated absolute acquisition delay for all heterogeneous sensing chains. Boldface distinguishes the present study from the previously published systems. Power consumption and battery operating duration are different quantities. LOS and NLOS denote line-of-sight and non-line-of-sight conditions, respectively.

**Table 2:** Configured acquisition frequencies and modality-specific ESP-NOW packet settings of the implemented system.

Node/Stream	Configured/Nominal Rate	Samples/Packet	Packet Size	Nominal Packet Interval
1/EMG1	2000 Hz	20	160 B	10 ms
5/EMG2	2000 Hz	20	160 B	10 ms
6/EMG3	2000 Hz	20	160 B	10 ms
2/PPG	400 Hz	16	196 B	40 ms
2/IMU	416 Hz	14	232 B	33.65 ms
3/Pinch	∼80 Hz	5	70 B	62.5 ms
4/Grip	∼80 Hz	5	70 B	62.5 ms

All the transmitted streams additionally include source-level timing and diagnostic information. Nominal packet intervals do not represent directly measured sensor-to-host delivery latency. Realized acquisition rates and sample continuity are reported separately in the Results section.

**Table 3:** Embedded firmware configuration, task scheduling, and queue capacities. Priorities correspond to FreeRTOS task priorities defined in the implementation.

Device	Firmware Source Revision	Task (FreeRTOS Priority)	Queue Capacity
ESP32-S3 gateway	v3.2 CRC Fix	USB TX (4); RX processing and sync scheduler in main loop	RX: 128; USB TX: 48
EMG1, EMG2, EMG3	v4	Acquisition (24); Sync (9); TX (8)	TX: 12; Sync: 4
PPG/IMU node	v6	Sensors (18); Sync (9); TX (8)	TX: 20; Sync: 4
Pinch/grip nodes	v2	Acquisition (20); Sync (9); TX (8); Status (3)	TX: 12; Sync: 4

Note: Numbers in parentheses indicate FreeRTOS task priorities. Higher values represent higher scheduling priority. RX and TX denote receive and transmit, respectively. Queue capacities indicate the number of items that each queue can hold. Gateway packet processing and synchronization scheduling are performed in the Arduino main loop rather than separately prioritized tasks.

**Table 4:** Full-system acquisition stability across three independent 30-min recordings. Index-based cadence and effective recorded rates were calculated using synchronized master timestamps and are reported as mean ± SD across runs. Packet delivery ratio, sample loss, and synchronization-valid coverage are pooled values.

Stream	Nominal	Index Cadence (Hz)	Recorded Rate (Hz)	Packet PDR	Sample Loss	Sync Valid
EMG1	2000 Hz	1999.903±0.001	1999.903±0.001	100.000%	0.000%	100.0%
EMG2	2000 Hz	1999.912±0.0002	1999.686±0.391	99.989%	0.011%	100.0%
EMG3	2000 Hz	1999.912±0.001	1999.830±0.122	99.996%	0.004%	100.0%
PPG	400 Hz	398.674±0.055	398.434±0.461	99.940%	0.060%	100.0%
IMU	416 Hz	414.765±0.015	414.506±0.463	99.938%	0.062%	100.0%
Pinch	∼80 Hz	85.139±0.039	85.139±0.039	100.000%	0.000%	100.0%
Grip	∼80 Hz	85.926±0.011	85.926±0.011	100.000%	0.000%	100.0%

**Table 5:** Recording-level and pooled external synchronization performance for the EMG2–EMG3 common-input experiment. The pooled statistics describe 897 accepted pulse events, while the three recordings are the independent units for between-run variability assessment.

Run	Timing Method	*N*	Skew (ms)	MAE (ms)	RMSE (ms)	P95 Abs. (ms)	Max Abs. (ms)	Trend (ms/min)
1	Initial offset	298	0.3427	0.3427	0.3749	0.5436	0.5670	0.1033
1	Drift compensated	298	−0.0222	0.0761	0.1346	0.2845	0.6503	0.0315
2	Initial offset	299	0.0999	0.1060	0.1342	0.2558	0.2998	0.0600
2	Drift compensated	299	0.0099	0.0733	0.1388	0.3267	0.6879	−0.0211
3	Initial offset	300	0.0563	0.0863	0.1040	0.1917	0.2082	0.0598
3	Drift compensated	300	0.0063	0.0767	0.1074	0.2128	0.4006	0.0048
Pooled	Initial offset only	897	0.1660	0.1780	0.2373	0.5079	0.5670	0.0743
Pooled	Drift compensated	897	−0.0019	0.0754	0.1277	0.2781	0.6879	0.0050

**Table 6:** Additional external clock-alignment validation with all six wireless nodes and all seven sensor streams active. The three recordings were separate acquisition intervals without power cycling the devices.

Recording	Valid Events	MAE (ms)	RMSE (ms)	P95 (ms)	Max (ms)
1	1770	0.3168	0.3885	0.7290	1.169
2	1775	0.3184	0.3896	0.7633	1.244
3	1774	0.3017	0.3682	0.6974	1.260
Pooled	5319	0.3123	0.3822	0.7272	1.260

**Table 7:** Pooled reference-event clock-alignment errors by physical sensor node across three recordings.

Node	Valid Events	MAE (ms)	RMSE (ms)	P95 (ms)	Max (ms)
EMG1 (1)	890	0.3135	0.3944	0.8121	1.234
PPG/IMU (2)	885	0.3543	0.4182	0.7610	1.025
Pinch (3)	892	0.2926	0.3539	0.6385	1.260
Grip (4)	892	0.2858	0.3600	0.6854	1.244
EMG2 (5)	872	0.3038	0.3811	0.7704	1.169
EMG3 (6)	888	0.3238	0.3826	0.6963	1.014

**Table 8:** Pairwise GPIO reference-pulse alignment across the 11 heterogeneous physical-node pairs with all seven sensor streams active. Signed bias is the first node’s mapped GPIO-event time minus the second node’s time. Each pair uses only shared gateway reference pulses; the event-level statistics are descriptive across three continuous-clock acquisition intervals.

Node Pair	Events	Bias (ms)	MAE (ms)	RMSE (ms)	P95 (ms)	≤1 ms (%)
EMG1–PPG/IMU	883	−0.3506	0.4983	0.6003	1.1012	92.30%
EMG1–Pinch	890	−0.2042	0.4196	0.5351	1.0616	93.71%
EMG1–Grip	890	−0.2742	0.4589	0.5836	1.1436	91.80%
EMG2–PPG/IMU	865	−0.0611	0.3468	0.4376	0.8858	97.69%
EMG2–Pinch	872	+0.0833	0.3459	0.4305	0.7970	97.94%
EMG2–Grip	872	+0.0169	0.3285	0.4168	0.8619	98.62%
EMG3–PPG/IMU	881	−0.1000	0.4014	0.5026	1.0350	94.32%
EMG3–Pinch	888	+0.0476	0.3479	0.4415	0.8473	97.30%
EMG3–Grip	888	−0.0221	0.3772	0.4665	0.8940	97.41%
PPG/IMU–Pinch	885	+0.1464	0.3579	0.4453	0.8728	98.42%
PPG/IMU–Grip	885	+0.0770	0.3158	0.4130	0.8848	97.06%

**Table 9:** Offline synchronization parameter sensitivity under a common steady-state evaluation interval (951 accepted-exchange opportunities across six nodes after excluding the first 64 accepted exchanges per node). Fit RMS and drift-update variation are the medians of six node-specific median values and characterize internal model behavior, not absolute timing accuracy. The bold row indicates the selected configuration.

Window	RTT Margin (ms)	Valid Fits/951	Median Fit RMS (ms)	Median Drift Change (ppm)	Median Fit Points
16	1.5	580	0.334	0.00	5.0
16	3.0	950	0.540	12.08	9.0
16	5.0	951	0.824	14.45	13.0
32	1.5	863	0.246	0.00	6.5
**32**	**3.0**	**951**	**0.600**	**2.72**	**16.5**
32	5.0	951	0.872	3.95	25.0
64	1.5	949	0.280	0.00	12.5
64	3.0	951	0.611	0.58	33.5
64	5.0	951	0.875	0.87	49.5

A reported drift change of 0.00 ppm does not imply zero clock error; for restrictive filters it can result from successive fits using an unchanged subset of observations. Counts exclude warm-up for all the configurations; values are not independent sensor-clock error measurements.

**Table 10:** Network-load validation at approximately 1 m line of sight. The full-system condition uses the three 30-min sustained-operation recordings.

Configuration	PDR	Sample Loss	Sync Valid	Sync-Fit RMS	Mean RTT
1 × EMG	100.000%	0.000%	100.0%	0.064 ms	3.04 ms
3 × EMG	100.000%	0.000%	100.0%	0.578 ms	4.94 ms
6 nodes/7 streams	99.987%	0.012%	100.0%	0.568 ms	6.16 ms

**Table 11:** Distance and obstruction performance under full-system operation. The 1 m reference uses the three 30-min sustained-operation recordings; the other conditions comprise three 5-min repetitions. NLOS denotes the 5 m separate-room condition with a 20-cm-thick concrete wall and a closed fire-protection door.

Condition	PDR	Sample Loss	Sync Valid	Sync-Fit RMS	Mean RTT
∼1 m LOS	99.987%	0.012%	100.0%	0.568 ms	6.16 ms
∼5 m LOS	99.913%	0.096%	100.0%	0.566 ms	6.50 ms
∼10 m LOS	96.398%	4.003%	100.0%	0.487 ms	7.10 ms
∼5 m NLOS	99.187%	0.872%	100.0%	0.550 ms	6.75 ms

## Data Availability

The experimental datasets supporting the findings of this study are available from the corresponding author upon reasonable request. The embedded firmware and host-application source code are not available for external distribution in view of the planned future commercialization of the developed system. Detailed hardware and software configurations, synchronization procedures, experimental protocols, and quantitative results are provided in the manuscript to support methodological transparency.

## Abbreviations

The following abbreviations are used in this manuscript:

ADC	Analog-to-Digital Converter
AFE	Analog Front-End
BLE	Bluetooth Low Energy
CRC	Cyclic Redundancy Check
EMG	Electromyography
ESP-NOW	Espressif Connectionless Wireless Communication Protocol
IMU	Inertial Measurement Unit
LOS	Line of Sight
MAE	Mean Absolute Error
MCU	Microcontroller Unit
NLOS	Non-Line-of-Sight
PDR	Packet Delivery Ratio
PPG	Photoplethysmography
RMS	Root Mean Square
RMSE	Root Mean Square Error
RTT	Round-Trip Time
sEMG	Surface Electromyography
SPI	Serial Peripheral Interface
TDMA	Time-Division Multiple Access
USB	Universal Serial Bus
